# Darwinian selection of host and bacteria supports emergence of Lamarckian-like adaptation of the system as a whole

**DOI:** 10.1186/s13062-018-0224-7

**Published:** 2018-10-26

**Authors:** Dino Osmanovic, David A. Kessler, Yitzhak Rabin, Yoav Soen

**Affiliations:** 1 Department of Physics, Bar-Ilan University, 52900 Ramat Gan, Israel; 2 NYU-ECNU Institute of Physics at NYU, Shanghai, 200062 China; 3 Department of Biological Chemistry, Weizmann Institute of Science, 76100 Rehovot, Israel; 4 Department of Physics, Massachusetts Institute of Technology (MIT), MA 02139 Cambridge, USA

**Keywords:** Host-microbiome interactions, Holobiont, Darwinian selection, Emergent adaptation, Lamarckian adaptation, Vertical and horizontal transmission, Population genetics

## Abstract

**Background:**

The relatively fast selection of symbiotic bacteria within hosts and the potential transmission of these bacteria across generations of hosts raise the question of whether interactions between host and bacteria support emergent adaptive capabilities beyond those of germ-free hosts.

**Results:**

To investigate possibilities for emergent adaptations that may distinguish composite host-microbiome systems from germ-free hosts, we introduce a population genetics model of a host-microbiome system with vertical transmission of bacteria. The host and its bacteria are jointly exposed to a toxic agent, creating a toxic stress that can be alleviated by selection of resistant individuals and by secretion of a detoxification agent (“detox”). We show that toxic exposure in one generation of hosts leads to selection of resistant bacteria, which in turn, increases the toxic tolerance of the host’s offspring. Prolonged exposure to toxin over many host generations promotes anadditional form of emergent adaptation due to selection of hosts based on detox produced by their bacterial community as a whole (as opposed to properties of individual bacteria).

**Conclusions:**

These findings show that interactions between pure Darwinian selections of host and its bacteria can give rise to emergent adaptive capabilities, including Lamarckian-like adaptation of the host-microbiome system.

**Reviewers:**

This article was reviewed by Eugene Koonin, Yuri Wolf and Philippe Huneman.

**Electronic supplementary material:**

The online version of this article (10.1186/s13062-018-0224-7) contains supplementary material, which is available to authorized users.

## Open peer review

Reviewed by Eugene Koonin, Yuri Wolf and Philippe Huneman (Institut d’Histoire et de Philosophie des Sciences et des Techniques, CNRS/Université Paris I Sorbonne). PH was nominated by Eric Bapteste. For the full reviews, please go to the Reviewers’ comments section.

## Background

Evolutionary adaptations are commonly thought to be driven by genetic mutations occurring on a timescale of many generations. Selection of individuals with rare beneficial mutations and transmission of these mutations across generations can then support adaptive evolution of the population. The exclusive focus on rarely occurring mutations has recently been expanded [1–7] to consider various forms of non-Mendelian inheritance, including: transgenerational epigenetic phenomena [8–11], genome editing and mobility [12, 13], niche construction [14] and transmission of symbiotic microorganisms [3, 5, 6, 15–19]. The case of symbiotic organisms may be of particular interest because of its broad relevance to animals and plants and the potential of host-microbe interactions to support adaptations that were traditionally considered impossible for hosts and bacteria on their own [3, 5, 17, 20, 21]. This is primarily due to a fundamental distinction between composite, host-microbiome systems and germ-free hosts, namely that the former undergo intertwined selections, operating on different timescales: rapid selections of symbiotic microorganisms within the host and slower selection of the host (with its bacterial population) [22]. While the selection of each bacterium is governed by its individual traits, selection of the host depends jointly on the traits of the host and the properties of its bacterial community [20, 23–28]. This community can vary during the lifetime of the host and can also be transferred across generations, as well as between neighboring hosts [29–35]. Whether symbiosis between a host and microorganisms (collectively referred to as a holobiont [17, 36]) warrants a significant change to evolutionary thinking is currently under debate [37–40]. In particular, it is not clear whether the association between host and bacteria is tight enough to consider the holobiont as a unit of selection in evolution [37, 38] and whether transmission of bacteria across generations of hosts is stable enough to support non-traditional adaptive capabilities. To investigate the feasibility of emergent adaptations, we introduce a modeling framework that avoids debated assumptions and relies instead on interactions between well-accepted Darwinian selections of host and resident bacteria. This allows us to study how general types of interactions influence the adaptation of host and bacteria on a wide range of timescales. Our modelling approach builds on the traditional framework of population genetics [41, 42], but extends it to account for important host-microbiome considerations that are not relevant for a population of germ-free hosts. In this model, we evaluate the adaptation of host and vertically-transmitted bacteria, which are jointly exposed to a toxic agent. The exposure promotes Darwinian selections that occur on different timescales for host and bacteria. We find that the combined effect of these selections has profound implications. Among these, we show that the interaction between the selections of host and bacteria can give rise to an emergent, Lamarckian-like adaptation of the host-microbiome system within a single host generation. This effect is mediated by distinct modes of stress alleviation and it has non-trivial dependence on the environmental conditions and the traits of the system. Persistence of the exposure over timescales longer than a host generation promotes additional selection of hosts containing bacterial communities, which secrete higher average detox per bacterium (in contrast to selection of individual bacteria, which takes place on much shorter timescales). This gives rise to a second mode of emergent adaptation that is independent of the Lamarckian-like adaptation within a host generation. In both cases, however, most of the adaptive benefit to the host is not attributable to changes in its own traits, but rather to alterations in its bacterial community. These alterations promote an increase in toxin tolerance which persists over periods longer than a host generation but shorter than typical evolutionary timescales of the host.

## Results

### General considerations of the model

We consider the simple case of a host-microbiome system in which every host is associated with a single species of bacteria that is transmitted to the host's offspring with perfect fidelity. We take the generation time of a host to be much larger than for bacteria and we probabilistically determine the survival of each host and bacterium, according to their state at the end of the respective generation time (as detailed below). Each surviving host and bacterium gives rise to one offspring that inherits the traits of its parent, subject to a small random modification depending on a constant mutation rate, ***μ*** (no epigenetics is considered). The host and its bacterial community are jointly exposed to a toxin of concentration ***T***, thus creating a stress that impacts the survival probability of the host and each of its bacteria. This stress depends both on their intrinsic traits and on how they interact with one another. To investigate whether and how the coupling between the survival of host and bacteria could support non-traditional modes of adaptation, we consider broadly applicable types of interactions between host and bacteria. The mathematical representations of these interactions was chosen to simplify the identification and analysis of general effects which apply to many host-microbiome systems (as opposed to a model designed to fit a specific system).

We start by defining the toxic stress experienced by individual host and bacterium. Since this stress depends on the level of toxin, ***T***, and on the individual’s sensitivity to the toxin, ***x***, we define the instantaneous toxic stress for host and bacteria as ***S***_***H***_ **=** ***x***_***H***_ ***T*** and ***S***_***B***_ **=** ***x***_***B***_ ***T***, respectively. Accordingly, this stress can be alleviated by cell-intrinsic reduction in sensitivity and/or by secretion of a detoxifying agent, “detox”, which reduces the toxic challenge (with or without associated cost).

Unlike in a germ-free system, a host in a composite host-microbiome system is influenced (and/or dependent) on bacterial-derived nutrients and various other factors [43–45]. Exposure to toxin may therefore lead to physiological stress to the host due to a significant loss of bacteria. An indirect stress to the host can also be induced by factors that promote a significant excess of bacteria. We model these effects by considering a physiological stress, ***S***_***ph***_, which depends on deviations from a “preferred” size of the bacterial population. From the bacterial perspective, on the other hand, the host provides a niche of a particular size (carrying capacity for bacteria). In the simpler case of free-living bacteria in a fixed environment, the carrying capacity is typically modelled by a constant parameter, representing the amount of extractable resources. The fixed niche assumption, however, does not necessarily hold when the bacteria are accommodated inside a host which can modulate the size of the niche under stress [23]. Since we do not know in advance whether and how a host’s stress influences the number of bacteria that can be accommodated, we constructed a population model in which this influence is determined by natural selection.

Altogether, the model considers host-microbiome interactions that are mediated by: (i) mutual alleviation of toxic challenge via secretion of a detoxification agent (“detox”), (ii) dependence of the hosts’ well-being on the size of the bacterial population and (iii) modulation of the bacterial niche size based on the toxic stress experienced by the host.

### Model formulation

For each host and bacterium, we assign a probability of survival to reproduction, ***P***_***H***_ and ***P***_***B***_, defined respectively as:

1 $$ {\boldsymbol{P}}_{\boldsymbol{H}}=\left(\mathbf{1}-{\boldsymbol{N}}_{\boldsymbol{H}}/\mathbf{2}{\boldsymbol{K}}_{\boldsymbol{H}}\right)\mathbf{\exp}\left[-\left({\widehat{\boldsymbol{S}}}_{\boldsymbol{H}}+{\widehat{\boldsymbol{S}}}_{\boldsymbol{P}\boldsymbol{h}}\right)\right] $$

2 $$ {\boldsymbol{P}}_{\boldsymbol{B}}=\left(\mathbf{1}-{\boldsymbol{N}}_{\boldsymbol{B}}/\mathbf{2}{\boldsymbol{K}}_{\boldsymbol{B}}\right)\mathbf{\exp}\left(-{\boldsymbol{S}}_{\boldsymbol{B}}\right) $$

Here, ***N***_***H***_ and ***N***_***B***_ are the population sizes of hosts and bacteria per host, respectively, ***K***_***H***_ is the maximal number of hosts that can be supported by the external environment (carrying capacity for hosts) and ***K***_***B***_ is the number of bacteria that can be accommodated in the host (carrying capacity for bacteria). The toxic and physiological stress to the host, ***Ŝ***_***H***_ **= <*S***_***H***_ ***>***_***t***_ and ***Ŝ***_***Ph***_ **=** ***ln*(<*N***_***B***_***>***_***t***_***/K***_***B***_^***0***^**)** + **(**1 **- <*N***_***B***_***>***_***t***_***/K***_***B***_^***0***^**)**, are defined respectively in terms of time averages of ***S***_***H***_ and ***N***_***B***_ over a host generation (interval between host reproduction events; recall that the probability of survival is calculated only at the end of each generation). The physiological stress vanishes when the time-averaged bacterial population, **<*N***_***B***_***>***_***t***_, reaches a size determined by the fixed parameter, ***K***_***B***_^***0***^. The latter also sets an inverse scale (***1/K***_***B***_^***0***^) for the negative impact of losing too many bacteria or having to support excess numbers of bacteria [44].

To test if selection might favor hosts that react to toxic stress by modulating the niche available for bacteria [46–49], we consider a population of hosts, each with a distinct dependence of the carrying capacity on the toxic stress of the host. For that, we define ***K***_***B***_ as:

3 $$ {\boldsymbol{K}}_{\boldsymbol{B}}={\boldsymbol{K}}_{\boldsymbol{B}}^{\mathbf{0}}\left(\mathbf{1}+\boldsymbol{\delta} \cdot {\boldsymbol{S}}_{\boldsymbol{H}}\right) $$

where **δ** is an evolvable trait, determining how the bacterial niche in the host is affected by the toxic stress it experiences. Since bacteria can affect this stress by secreting detox on a timescale shorter than a host generation, ***K***_***B***_ is jointly influenced by the host and the bacteria. To enable unbiased analysis of how ***K***_***B***_ changes in response to selection under exposure to toxin, we considered a starting population of hosts with a broad distribution of **δ**’s, symmetric around zero.

We assume that all the hosts and their bacteria are exposed, at time ***t***, to the same influx of active toxin, ***θ(t)***, applied instantaneously (i.e., in one bacterial generation, ***Δt***). This toxin can be neutralized by release of detox from the host and each of its bacteria [44, 49–51]:

4 $$ \boldsymbol{T}\left(\boldsymbol{t}+\boldsymbol{\Delta} \boldsymbol{t}\right)=\boldsymbol{T}\left(\boldsymbol{t}\right)\ \mathbf{\exp}\left(-{\boldsymbol{\uplambda}}_{\mathbf{B}}\mathbf{\sum}{\mathbf{y}}_{\mathbf{B}}-{\boldsymbol{\uplambda}}_{\mathbf{H}}{\mathbf{y}}_{\mathbf{H}}\right)+\boldsymbol{\theta} \left(\boldsymbol{t}\right) $$

where ***y***_***H***_ and ***y***_***B***_ are the instantaneous amounts of detox secreted inside the host (by resident bacteria and the host itself) and **λ**_***H***_ and **λ**_***B***_ are the respective detoxification capacities of host and bacteria. We assume that all the bacteria of a given host benefit equally from the total amount of detox, regardless of their individual contributions to this total detox. The effect of having a cost associated with the secretion of detox by the bacteria is investigated in an extended version of the model (Additional file 1).

The evolvable traits of the model (***x***, ***y*** and **δ**) are initially drawn from trait-specific distributions and are modified by the joint actions of mutation and selection. Surviving bacteria divide at every time step of the simulation (***Δt***), while the surviving hosts reproduce every **τ** generations of bacteria (so that the host generation time is **τ** ***Δt***). We consider the simplest reproduction model in which each of the surviving hosts and bacteria gives rise to one offspring that inherits the traits of its parent, subject to a small random modification depending on a constant mutation rate, ***μ***:

5 $$ {\boldsymbol{Z}}_{\mathbf{offspring}}={\boldsymbol{Z}}_{\mathbf{parent}}+\boldsymbol{\eta} \mathbf{\surd}\boldsymbol{\mu } -{\boldsymbol{\beta}}_{\boldsymbol{z}}\boldsymbol{\mu} \left({\boldsymbol{Z}}_{\mathbf{parent}}-{\boldsymbol{Z}}_{\mathbf{0}}\right) $$

Here ***Z*** corresponds to any of the evolving traits ***x***, ***y*** and **δ**, ***η*** is a standard Gaussian deviate with zero mean, and the parameters, ***Z***_**0**_ and ***β***_***z***_, are trait-specific coefficients controlling the peak and width of the steady state distributions (specified in Methods). Note that 1/***β***_***z***_ sets a characteristic time for the distribution of a trait **Z** to return to steady state, following an initial perturbation. The values of ***β***_***y***_ and ***β***_***δ***_ were chosen to support broad distributions of ***y*** and **δ**, respectively. To prevent a trivial solution in which all the individuals are completely insensitive to toxin, the sensitivity distribution (for ***Z*** *=* ***x***_***H***_ and ***x***_***B***_) is truncated at ***x*** = 0. We also avoid negative values of detox secretion by setting negative ***y*** values in Eq. 5 to zero. The remaining dynamic variables are updated in every generation of bacteria (***N***_***B***_, ***T***, ***S***_***H***_, ***S***_***B***_ and ***K***_***B***_) and host (***N***_***H***_). The current study was based on an initial population of 32000 hosts (***N***_***H***_ ***= K***_***H***_ = 32000) with 100 bacteria per host (***N***_***B***_ ***= K***_***B***_^***0***^ = 100). The host generation time was set to **τ =** 100 bacterial generations and all the mutation rates were ***μ*** = 10^− 3^ per generation (for both host and bacteria).

### Stress-dependent adjustment of bacterial niche size

**Fig. 1:**
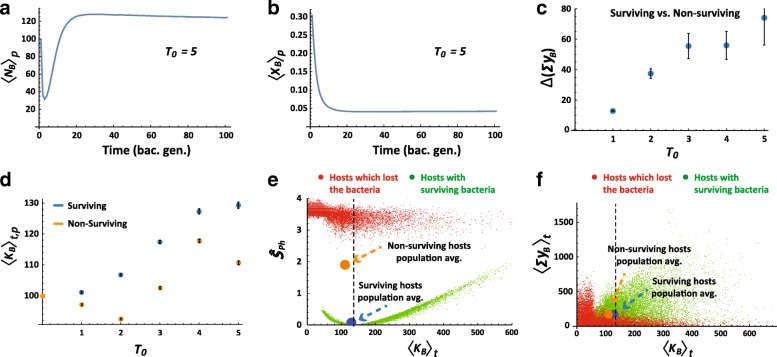
Stress-dependent adjustment of bacterial niche size. **a, b** Short term kinetics of the population-averaged number of bacteria, <***N***_***B***_ >_***P***_ (**a**) and bacterial sensitivity, <***x***_***B***_ >_***P***_ (**b**) for hosts which survived a single pulse of exposure to toxin, ***T***_***0***_ = 5, applied at the initial time step. **c** Average difference ± standard error (SE) between surviving and non-surviving hosts with respect to the total amount of detox secreted by bacteria over a host generation (shown for each ***T***_***0***_). **d** Mean carrying capacity for bacteria in the population of hosts, averaged (± SE) over a host generation at different levels of ***T***_***0***_. **e** Average physiological stress over a host generation, ***Ŝ***_***Ph***_, versus the time average of its carrying capacity for bacteria. Green and red points represent hosts with surviving and non-surviving bacteria, respectively. Blue and orange circles mark population averages for surviving and non-surviving hosts, respectively. Dotted line marks carrying capacity which minimizes the physiological stress. **f** Same as (e) for the time average of total bacterial detox versus bacterial carrying capacity. Time and population averages are denoted by ***t*** and ***p*** subscripts, respectively)

We examined the effects of exposure to a single pulse of toxin, ***T***_***0***_, applied at ***t***_***0***_ (i.e. ***θ(t***_***0***_*) =* ***T***_***0***_). On timescales smaller than one host generation (100***Δt***), the bacterial community undergoes selection for less sensitive bacteria, accompanied by a drop in the bacterial population size (Figs. 1a,b). In a system with only one level of selection (e.g. free-living bacteria), this would be the only adaptive change. However, when the bacterial population is symbiotically coupled to a host, the survival of each host and bacterium depends also on the amount of detox secreted by the bacteria (Fig. 1c). The secretion is higher for hosts which react to the toxic stress by increasing their carrying capacity for bacteria (i.e. hosts with **δ** > 0; Additional file 1: Figure S1A). This leads to stress-dependent selection of hosts which provide a larger bacterial niche ***K***_***B***_ (Fig. 1d), thus increasing the number of resistant bacteria beyond ***K***_***B***_^***0***^ (Fig. 1a). The benefit from this increase is two-fold: Alleviation of the negative impact of losing bacteria (by assisting recovery of the bacterial population; Fig. 1e, Additional file 1: Figure S1B) and elevation of the total amount of secreted detox (Fig. 1f, Additional file 1: Figure S1C). However, when **<*N***_***B***_***>***_***t***_ is larger than ***K***_***B***_^***0***^, the benefit from higher detox secretion is counteracted by the negative impact of bacterial overload. The overall effect of these positive and negative factors adjusts the bacterial population size in a stress-dependent manner which tends to maximize the probability of survival of the host.

### Stress-dependent adaptation within a host generation

**Fig. 2:**
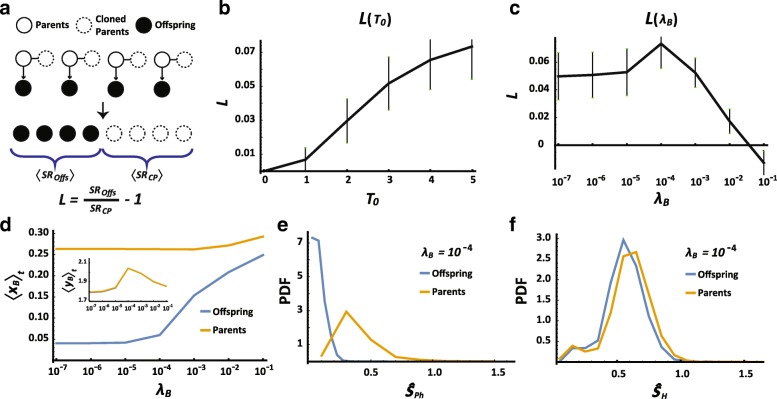
Stress-dependent adaptation within one host generation. **a** Schematics of the Lamarckian evaluation procedure. **b, c** The *Lamarckian* as a function of toxic exposure (**b**) and bacterial detox coefficient (**c**). **d** Bacterial sensitivity and detox per bacteria (inset) as a function of bacterial detox coefficient, after exposure to toxin (***T***_***0***_ = 5). Shown are time (and population) averages over one generation of unexposed ‘clones’ of surviving parents (orange) and their offspring (blue). **e, f** Distributions of physiological (***Ŝ***_***Ph***_) and toxic stress (***Ŝ***_***H***_), experienced by cloned parents and their offspring, following exposure to a toxin pulse (***T***_***0***_ = 5), applied at the initial time step. Shown for the case of **λ**_***B***_ = 10^− 4^

Microbiomes that are modified by the stress in one host generation can be transmitted to the host’s offspring, potentially increasing its stress tolerance. In order to evaluate the possibility and magnitude of this outcome, we introduce a new measure, termed the “*Lamarckian*”. It quantifies the change in the survival probability of the host's offspring due to (stress-dependent) microbial variations that were induced during the lifetime of the parental host. To take into account only those changes that were induced by the environmental stress, we compared the survival of offspring hosts to the survival of their parents as determined by the initial state of these parents. To implement this analysis in the simulation, we identify the hosts which survived a generation of exposure, revert them to the initial state of their microbiome and apply a new simulation to the reverted hosts (denoted “cloned parents”) and their offspring (Fig. 2a). We then compare the survival rates of the offspring (***SR***_***Offs***_) to that of their cloned parents (***SR***_***CP***_) and define the *Lamarckian*, ***L***, as:

6 $$ \boldsymbol{L}={\boldsymbol{SR}}_{\boldsymbol{Offs}}/{\boldsymbol{SR}}_{\boldsymbol{CP}}-\mathbf{1}, $$

so that it is positive if the average survival increases due to transfer of changes acquired during the previous host generation. The use of the initial state of the parental host and its microbiome allows us to distinguish the increase of tolerance due to selection of initially better fit parents, from the gain of tolerance due to transmission of changes acquired during a host generation (not present in the initial parental clones).

For a given **λ**_***B***_, we find that ***L*** is an increasing function of the injected amount of toxin, vanishing only at low ***T***_***0***_ (Fig. 2b). For a given ***T***_***0***_, on the other hand, the *Lamarckian* has a non-monotonic dependence on **λ**_***B***_. This is manifested by a nearly constant ***L*** > 0 over a range of small **λ**_***B***_, followed by an increase to a maximum at intermediate values of **λ**_***B***_ and lastly, a decline at sufficiently large **λ**_***B***_ (Fig. 2c). 

The positive *Lamarckian* is the result of transgenerational transfer of a bacterial population that was selected for lower toxin sensitivity during the parental host generation (Fig. 2d). To determine how these bacteria increase the probability of survival of the hosts’ offspring, we analyzed the toxic and physiologic stress in the offspring vs. their cloned parents. For small enough **λ**_***B***_, the benefit from bacterial secretion of detox is negligible and the positive *Lamarckian* is primarily due to alleviation of the physiological stress in the offspring (Additional file 1: Figure S2). This is due to inheritance of bacteria that are less sensitive to toxin (Fig. 2d), so that the population size of bacteria in the exposed offspring remains closer to the preferred value (***K***_***B***_^***0***^) compared to the bacterial population size in their cloned parents. At intermediate values of **λ**_***B***_, the offspring have an additional benefit due to the detox secreted by their toxin-resistant bacteria, thus making a second contribution to the *Lamarckian* (Fig. 2e,f). However, when **λ**_***B***_ is large enough to support substantial neutralization of toxin during a single host generation (Additional file 1: Figure S3), the selection pressure on both hosts and their microbiomes is weakened and the *Lamarckian* decreases because of the diminished difference between parents and offspring (Additional file 1: Figure S2B).

### Selection of hosts based on collective traits of their bacterial community (‘*Microbiome selection*’)

**Fig. 3:**
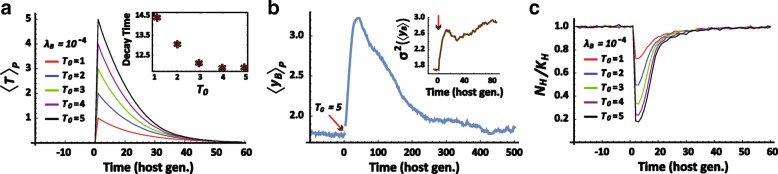
Stress-dependent selection of hosts, based on microbiome properties. **a** Temporal kinetics of active toxin for different initial levels of toxin, ***T***_***0***_. Inset displays the time to neutralize 50% of the toxin. **b** Temporal kinetics of average detox secretion per bacteria following exposure to toxin at ***T***_***0***_ = 5 (red arrow). **λ**_***B***_ = 10^− 4^. Inset reveals an increase of inter-hosts variance in average detox per bacteria. **c** Kinetics of host population size, ***N***_***H***_, normalized by the host carrying capacity, ***K***_***H***_

When the toxic pressure persists over timescales larger than one host generation (Fig. 3a), the selection favors hosts with bacterial communities that secrete higher amounts of detox per bacterium, <***y***_***B***_ >_***P***_ (Fig. 3b). Since this selection is determined primarily by the microbiome as a whole and not by individual bacteria, we will refer to it as *Microbiome selection*. When the secretion of detox comes at a cost to the individual, the microbiome selection for detox is weakened, but it is still apparent over a broad range of cost levels (Additional file 1: Figure S4A,B). The negative effect of the cost on the survival probability of bacteria (Supplementary Information, Eq. 2′) aggravates the initial loss of bacteria and increases the physiological stress to the host (Additional file 1: Figure S4C). This promotes selection of hosts that can partially alleviate this stress by accommodating larger numbers of bacteria (Additional file 1: Figure S4D). The cost on bacterial detox therefore strengthens the selection of hosts which accommodate more bacteria at the expense of weakening the selection for increased detox per bacterium. The Lamarckian effect, on the other hand, is not compromised by the cost of detox (Additional file 1: Figure S5A,B), because the increase of physiological stress in parental hosts is larger than the corresponding increase in their offspring (Additional file 1: Figure S5C).

In the current model, *Microbiome Selection* occurs only at the time of host reproduction. If the toxin persists over a period longer than ***μ***^***− 1***^ bacterial generations and the elimination of mutations is sufficiently slow (i.e. small enough ***β***_***y***_), the selection is accompanied by significant accumulation of bacterial mutations. Such accumulation enhances the selection for higher <***y***_***B***_>, thus increasing the detoxification rate (Fig. 3a, inset) and expediting host adaptation (Fig. 3c). This is accompanied by extended persistence of high detox levels (Fig. 3b) and by elevated detox variability across host-microbiome systems (Fig. 3b, inset). Additional increase of variability under stress is observed in the carrying capacity for bacteria and in the size of the bacterial population (Additional file 1: Figures S6A,B).

**Fig. 4:**
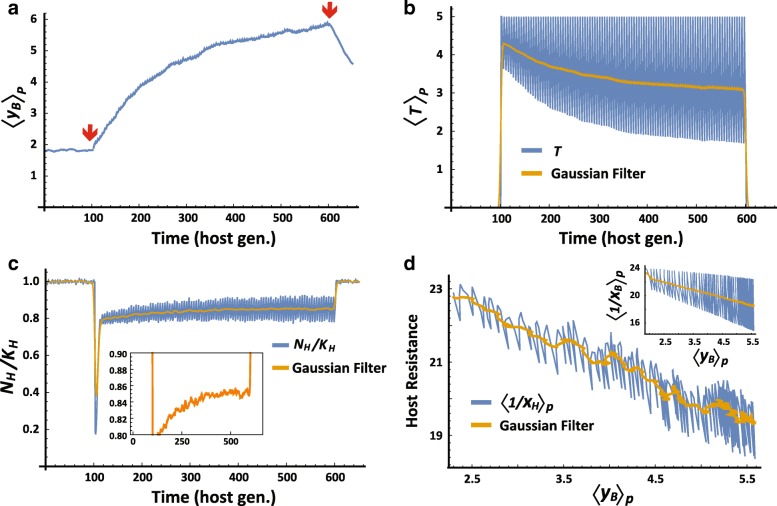
Multi-generational coupling between microbiome properties and host-intrinsic traits. The population of host-microbiome systems was subjected to successive resetting of the active toxin to ***T*** = 5, every 5 host generations. **a-c** Temporal kinetic profiles of average detox per bacteria (**a**), active toxin (**b**) and normalized size of the host population (**c**), with a magnified scale in the inset. Red arrows in (**a**) mark the start and end of the successive resetting of the toxin. **d** Inverse correlation between the increase in detox secretion per bacterium and the average toxin resistance (inverse sensitivity) of host, **1/*x***_***H***_, and bacteria, **1/*x***_***B***_ (inset). Orange overlays correspond to Gaussian filtering of the measured properties

Following the neutralization of toxin, the selected bacterial mutations persist over a characteristic timescale of 1/***μ*** = 10 host generations, thus providing a ‘memory’ of the previous exposure. To evaluate the influence of this ‘memory’ on the tolerance to new exposures, we analyzed the response to repeated pulses of injected toxin, separated by time intervals shorter than 10 host generations. These re-exposures led to repetitive microbiome selections occurring at a rate that is sufficient to oppose the relaxation of <***y***_***B***_>_***P***_ to its (lower) equilibrium value (Fig. 4a vs. Fig. 3b). The resulting enhancement of detoxification (Fig. 4b), reduced the selection pressure on the host (Fig. 4c) and enabled the survival of intrinsically less resistant hosts and bacteria (Fig. 4d). Progressive reduction in the intrinsic resistance of the host due to repetitive selections of higher bacterial detox, is reminiscent of *Genetic Assimilation* by successive selections of host-intrinsic alleles [52, 53]. In the case of *Microbiome selection*, however, the gradual change in the population of hosts is caused by recurrent selection of variations in the bacterial population (analogously viewed as *“Bacterial Microbiome Assimilation”*). Bacterial variations emerge on faster timescales compared with germline mutations in the host genome, but they are considerably less stable than host-intrinsic mutations. Nonetheless, when the repertoire of host-intrinsic alleles available for selection is limited, the hosts’ population may become more strongly dependent on variations that emerge within the host’s lifetime (e.g. bacterial and epigenetic variations).

### Potential strategies for *Lamarckian* estimation in experimental settings

Quantification of the *Lamarckian* in the model was done by reverting a subset of host-microbiome systems to their initial state and re-subjecting them to toxin. Since we cannot apply this procedure to experimental data, the *Lamarckian* of a real system would need to be approximated by other means, which may be context-dependent. In organisms such as flies and worms, where the bacteria can be removed without a significant impact on survival (e.g. by egg dechorionation followed by rearing on a sufficiently rich diet [54–56]), the *Lamarckian* can be approximated in steps that are conceptually similar to the simulation procedure: first, the hosts are exposed to a challenge and their offspring are cleared of bacteria and separated into two subpopulations. One of these subpopulations is re-colonized with (‘naïve’) microbiota from untreated hosts (as in refs. [35, 57]), while the other is colonized by (‘experienced’) microbiota from a group of hosts which survived exposure to a challenge. The *Lamarckian* would then be estimated from the ratio between the survival rates of hosts with experienced vs. naïve microbiota (i.e. ***L*** ≈ ***SR***_***Exp. microb.***_ ***/ SR***_***Naïve microb.***_ ***- 1***). This evaluation, however, neglects other types of changes that may have been acquired and transmitted to offspring (e.g. transfer of small RNAs [10], altered deposition of maternal RNA [58], persistent chromatin modifications [8], horizontal transfer of biochemical signals [59] and/or other modes of local niche construction [14]). Additional consideration that may affect the evaluation is horizontal transmission of bacteria to bystander hosts and/or to offspring of other hosts. The above effects can be taken into account by removing the bacteria from two untreated subpopulations, re-colonizing hosts with ‘naïve’ and ‘experienced’ microbiota, respectively, and estimating the *Lamarckian* from the survival of these colonized populations under challenge. More generally, it should also be possible to obtain a relative measure of the *Lamarckian* by manipulating the microbiome (or any other factor) in a subpopulation of hosts and evaluating the relative difference in offspring adaptation compared to offspring of non-manipulated parents (taken from the same distribution of hosts).

## Discussion

We explored adaptation dynamics in a host-microbiome model in which Darwinian selection of the host is coupled to a faster selection of its vertically-transmitted bacteria. It is generally accepted that selection of bacteria occurs in every animal and plant and that some of these bacteria can be horizontally and/or vertically transmitted [26, 60, 61]. Transmission of a bacterial population that has acquired changes during a single host lifetime can potentially alter the state of the host and may confer adaptive capabilities that are traditionally considered impossible for germ-free hosts and free-living bacteria. Rigorous evaluation of these capabilities has been hampered, however, by disagreement about how to conceptualize the adaptation and evolution of a composite system of host and bacteria [26, 37–39]. In particular, it is not clear whether the association of the bacterial community with a host (and its offspring) is tight enough to support their co-adaptation and evolution as a (holobiont) unit. Our model bypasses this difficulty by relying on well-accepted Darwinian selections operating, respectively, on hosts and (vertically transmitted) bacteria. We show that interaction between these selections can give rise to previously unrealized modes of emergent of adaptation, promoted by bacterial influence on the survival probability of the host. This includes a gain in offspring tolerance due to toxic exposure of the parental host (Lamarckian effect) and selection of hosts based on a collective property of their bacterial community (Microbiome selection). This was evidenced, for example, by a progressive increase in the host population size (Fig. 4c) despite a reduction in the intrinsic resistance of the host (Fig. 4d).

Within the simplified model in which the survival of the host is evaluated only at the time of reproduction, Lamarckian adaptation arises due to rapid selection and transmission of resistant bacteria. This transmission alleviates the loss of bacteria following toxic exposure and confers two types of benefits to the host’s offspring: a) reduction of physiological stress and b) increase in the total detox secreted by the bacteria. The contribution of each of these effects to Lamarckian-like adaptation depends on the level of toxic exposure and the detoxification capacity. Selection of hosts with higher bacterial detox, on the other hand, occurs on a timescale larger than one host generation and therefore cannot contribute to the *Lamarckian* which measures the offspring’s gain in tolerance due to changes that occurred within a single generation of parent hosts. *Microbiome selection* is nonetheless the main contributor to the progressive increase in tolerance over multiple host generations. Taken together, the Lamarckian adaptation is mediated by selection of resistant bacteria within one host generation while the longer-term adaptation under prolonged toxic pressure is achieved by selection of bacterial communities with higher detox per bacterium.

Although the aforementioned capabilities are linked to common features of host-microbiome systems, the scope and generality of the current model are limited by its simplifying assumptions. Studying the effects of factors that are not included in the present work (e.g. multiple species of symbionts and/or pathogens, epigenetic effects, etc.) requires suitable extensions of the model. A noteworthy aspect that is not covered in our model is the potential effect of horizontal transmission of bacteria. While the latter is generally expected to erode specific associations between host and bacteria [62], theoretical analysis of horizontal transfer under selection has demonstrated the feasibility of interspecific epistasis effects even in the absence of perfect transmission [63]. This possibility is further supported by evidence of high interpersonal variability in the composition of microbiota in different body habitats [64–66], as well as by dependence of the microbiome composition on genetic determinants of the host [66, 67] and other host-specific factors [68, 69]. Based on the theoretical prediction and the experimental findings (as well as the insights from our simulations), we expect that the selection for higher bacterial detox will be weakened by horizontal transmission, but will vanish only in the limit of strong “mixing” (i.e. when all the hosts in a given generation are populated with indistinguishable bacterial communities). Emergent Lamarckian adaptation, on the other hand, should hold even in the extreme case of complete bacterial mixing, because it is mediated by rapid selection of resistant bacteria followed by transfer to the next generation of hosts. Horizontal transfer is not expected to compromise the acquisition of toxin tolerance, but rather to promote sharing of the benefits with offspring of other hosts.

The large timescale separation between the selection of individual resistant bacteria and selection of bacterial communities which secrete more detox, reflects a lack of mechanism (in our model) for changing <***y***_***B***_ > during a single host generation (with the possible exception of rare cases of rapid changes in <***y***_***B***_ > due to amplification of very small numbers of resistant bacteria). Selection for higher bacterial detox within the lifetime of the host may nonetheless be possible if the stress of the host influences the distributions of its bacterial phenotypes. While we did not consider this type of influence in our simplified model, it likely applies to every host-microbiome system (due to the numerous possibilities for 2-way interactions between the host and its symbionts). An extension of the model which allows the stress of the host to influence the bacterial distribution of detox (e.g. by subjecting ***β***_***y***_ to stress-dependent dynamics similar to that of ***K***_***B***_) may therefore support additional adaptation due to increased secretion of bacterial detox during the host's lifetime. This could allow the host to benefit from newly-forming bacterial mutations and may further affect the *Lamarckian*.

Finally, we would like to re-emphasize that the proposed modelling framework does not aim to fit a particular host-microbiome system, but rather to investigate the possible modes of adaptation in a system with interactions between coupled selections of host and vertically transmitted bacteria. We show that such interactions can support non-traditional adaptive modes, including a gain in tolerance of the host’s offspring due to toxic exposure of its parent, and longer-term selection of hosts based on collective detox secretion by their bacterial communities. When the toxic challenge persists, or is frequently re-encountered, the recurrent selection of detoxifying microbiomes leads to further reduction of toxic pressure on the host and weakens the selection of hosts with higher intrinsic resistance.

## Conclusions

Our findings show that interactions between pure Darwinian selections of host and its bacteria can give rise to emergent adaptive capabilities, including Lamarckian-like adaptation of the host-microbiome system. Since the model considers general factors that are typical of host-microbiome systems, the emergent capabilities are likely relevant to most animals and plants as well as to other types of organizations, which satisfy the general assumptions of this modelling framework. The latter can be readily adjusted to incorporate additional factors, such as having multiple species of symbionts and pathogens (with inter-species competition and/or cooperation), asynchronous reproduction modes, epigenetic effects, ecological influences, horizontal transfer of bacteria (and/or toxin) between hosts and more.

## Reviewers’ comments

### Referee report 1: Eugene Koonin

Osmanovic and colleagues describe an agent-based model of host-microbiome coevolution. Within the framework of the model, they show that, when the evolution of both the host and the microbiota are modeled under standard population-genetic (“Darwinian”) assumptions, Lamarckian-type adaptation appears as an emergent phenomenon. Specifically, as a result of the exposure to a toxic agent, the holobiont acquires specific resistance to this particular toxin. Feasible experiments to test the predictions of the model are described.

I think this is a very good paper, the model is simple, elegant and well described. I do not see any specific flaws. My only suggestion is to make a special section for the proposed experimental validation of the model, so that the reader is immediately aware of the detailed description of such possible experiments.

**Author response:** *We appreciate the reviewer’s suggestion. The revised manuscript dedicates a section to proposed strategies for estimating the Lamarckian in real experimental settings.*

### Referee report 2: Yuri wolf

Osmanovic et al. present a model for host adaptation for the toxic stress that is facilitated by the adaptation of its microbial symbionts. Due to the microbial generation time being much shorter than the host generation time, Darwinian adaptation of the microbial community, taking many generations of symbionts, appears to be rapid in the host timescale (i.e. occurs much faster than the host generation time). This creates an illusion of Lamarckian evolution whereby the adaptive change in the host microbiome is passed to the host’s offspring. In my opinion the model is reasonably realistic, but the effect that it describes is rather obvious. The authors themselves note that the timescale separation is the key (“The exposure promotes Darwinian selections that occur on different timescales for host and bacteria”). At the host population time scale the adaptation is practically instantaneous, although it takes multiple generations art the symbiont time scale. If individual hosts do not exchange their symbionts (the least realistic assumption of the whole model), at much longer time scales hosts compete with each other on the grounds of having more beneficial microbiomes. I believe that the authors somewhat overstate the emergent nature of the of the host-level effect. The distinct “macroscopic” phenomena (fast and heritable adaptation of an individual host) do emerge from the “microscopic” action (Darwinian adaptation in the symbiotic microbial population), but the effect is rather straightforward (compare with the emergence of enzymatic properties of a protein from the quantum-mechanical interactions of the constituent atoms, a collective phenomenon that is not easily [if at all] deducible from the microscopic level). The detailed analysis of the model dynamics might be of interest to readers working on similar problems.

**Author response:** *We thank the reviewer for these comments.*

### Referee report 3: Philippe Huneman

This paper proposes a model of host-symbiont evolution in response to toxins as a way to explain the possibility of “emergent Lamarckian-like adaptations”. The key feature of the selection process underwent by symbionts and bacteria is the timescale difference between response to selection in one and the other side. The emergence of the adaptation to toxin as an effect of selection on the host-symbiont system may arise in a single host generation and therefore appear as a Lamarckian style adaptation of the host to its new environment. To some extent this paper can be read as a demonstration, based on a model simulation, that some seeming Lamarckian processes are in fact Darwinian when they are decomposed into their component-processes. Such reading contrasts with a claim made elsewhere (line 290) by the authors, according to which the paper contributes to the exploration of “non-traditional modes of adaptation”. There is an ambiguity here - and, to solve it, I think that what is shown is that there is one way to adaptation, namely natural selection, but that many subkinds of selective processes produce very distinct ‘styles’ of adaptation. I’ll go on by describing what according to me, constitutes the contribution of the paper to our conception of natural selection, and then raise two issues that I’d have liked the authors to engage. Given that the paper is explicitly conceived as a general model (rather than a realist or a precise model, according to Levins (1966) classical typology of epistemic goals) (p.5 line 109), I take it that the model’s contribution should be a novel understanding of some aspects of the adaptation process generalities; knowing which systems do satisfy this model is another question, which calls for other, experimental, investigations. First, the paper provides an interesting shift from the usual discussion regarding microbiomes with respect to selection. Most of the debates consider whether or not microbiomes with hosts, or holobionts as we now say, can be units of selection (e.g. Moran and Sloan 2011, etc). However, in the present model, the microbiome and the hosts are not a single unit of selection; on the contrary, what makes the novel style of adaptation possible is that there are two selective processes, on the host and on the symbiont. Thus the conceptual importance of microbiomes for evolutionary biology is not only that it challenges the traditional view of units of selection, but also that it diversifies the types of Darwinian adaptive processes. I take it as a significant achievement of the authors’ model.

Second, historically the paper can be viewed as part of a history of deflating lamarckian claims in evolutionary biology. While ‘Lamarckian’ means an explanation of adaptation referring to a process of adaptive variation, a thread in evolutionary biology consists in the exhibition of some Lamarckian process or feature, followed by the elaboration of an answer showing which non-trivial selective process can produce such seemingly Lamarckian feature. For instance the SOS stress system in bacteria, which increases the mutation rate in response to stress, was initially taken as Lamarckian but then proved to be plainly Darwinian, because the system does not genuinely produce an adaptive variation in reaction to its new environment. Thus the present paper can be seen as a novel episode in the long-lasting story of making apparent Lamarckian features into Darwinian selection. Its originality consists in tracing back the Lamarckian feature to the timescale difference between host and bacteria. Thus, a general lesson of such model consists in highlighting the crucial importance of timescale difference in evolutionary processes. Indeed, in the same vein we just proposed that thinking in terms of time scale differences casts a light on phenomena of epigenetic inheritance and includes them within a Darwinian framework (Danchin et al. Forth.), so that the whole inheritance system can be thought as adaptation in a neo-Darwinian way. This key role of timescales is often neglected when one discusses the possible challenges to the orthodox Darwinian view (non-genetic inheritance, some cases of phenotypic plasticity, etc) and it might be that considering such timescale difference makes the Darwinian evolutionary processes more complex and then more likely to account for their apparent counter examples. That would be the second general consequence of the paper, and it may concerns the debates on holobionts just mentioned, since some evolutionary consequences of the timescale difference between host and symbiont may be washed out when one subscribes to the concept of holobiont (and especially as a unit of selection). Then, two questions came to my mind regarding the paper.

The paper refers to the size of the host population when it discusses selection of hosts based on traits of their bacterial populations. It says that the benefit from the rapid variation in the bacterial population are high when the host population is small (line 272) - however, shouldn’t one expect that drift swamps selection in this case, so that host selection is not reliably conducive to adaptations?

The second question doesn’t concern the model proper, but the scope of the findings. Are there known systems that indeed feature lamarckian like adaptation and would in turn be explained by this model? Is it likely that well known host-microbiome systems such as squid-Vibrio Fischeri instantiate the present model? More generally could we already have an idea of the significance of such model for the actual biological world as we know it? Then one could view the cases of those Lamarckian-like evolution in a phylogenetic perspective, and therefore question what role played this type of adaptation in the history of life. References. Danchin E, Huneman P, Pocheville A (Forthcoming, 2018). “Early in life effects and the concept of heredity; reconciling neo-Darwinism with neo-Lamarckism under the banner of the Inclusive Evolutionary Synthesis” Philosophical Transactions of the Royal Society B. Forthcoming, 2018. Moran NA, Sloan DB (2015) “The Hologenome Concept: Helpful or Hollow?” PLoS biology, 13(12):e1002311.

**Author response:** *We appreciate the thoughtful comments and made a few text revisions to clarify some of the points.*

In addition, we would like to note that:

1. The emergent adaptation in the proposed model is indeed based on coupled Darwinian selections occurring on different timescales. In fact, one of the goals of considering pure Darwinian selection was to demonstrate that its combination with different timescales is sufficient to support Lamarckian adaptation as an emergent capability. The simplified model in this model was formulated in a manner which does not enable the host-microbiome system to benefit from newly-forming variations (emergent adaptation within generation is based on selection of existing variations). This, however, does not exclude the possibility that alternative formulations of coupling between host and bacteria would support adaptation by variations that are induced (or emerge spontaneously) within the lifetime of an individual host. A conceptual basis for such adaptation is described in Soen et al., *Biol. Direct* 2015 [6] and has been partially supported by a mathematical analysis that should be valid for any type of an individual, be it a single cell, animal or plant (Schreier et al. *Nature Comm*., 2017) [70].
2. We probably should have better explained our comment about the benefit from rapid variation when the host population is small (line 272 of the previous version). Our intention was to point out that in small populations of hosts, the relative extent of assimilation of phenotypes due to successive selections of mutations in the host’s microbiome (“*Bacterial Assimilation*”) increases in comparison to assimilation based on host-intrinsic genetic changes (“*Genetic Assimilation*”). This is because the repertoire of host-intrinsic alleles is likely to be more severely reduced compared with the repertoire of bacterial mutations which emerge on faster timescales. We clarified this point in the revised manuscript and we thank the reviewer for his comment.
3. It is important to make a clear distinction between Lamarckian adaptation (which takes place within a host generation) and longer term evolutionary implications of ongoing Lamarckian processes. In the current work we focused only on identifying emergent modes of adaptation that are made possible by coupling between selections occurring at different timescales. The wide range of model parameters which still admit Lamarckian-like adaptation, suggests that the accumulated outcomes of such emergent adaptation indeed feeds into evolutionary processes (along with other factors). How these adaptive capabilities may affect the evolutionary trajectory of a given species is, however, beyond the scope of this work.

## Methods

### Simulation procedures

The simulation starts with a population of hosts, each carrying a population of 100 bacteria. Host and bacterial properties (phenotypes) are initially drawn from defined distributions (steady state of Eq. 5 without toxin) with parameters: ***x***_***0***_ = 0.25, ***β***_***x***_ = 10, ***y***_***0***_ = 0, ***β***_***y***_ = 0.1, **δ**_***0***_ = 0 and ***β***_***δ***_ = 0.1.

In every time step of the simulation (one bacterial generation), each bacterium reproduces if its survival probability (Eq. 2) is larger than a random number (between 0 and 1) drawn from a uniform distribution. Each of the surviving bacteria (parents) persists at its current state and gives rise to a modified bacterium (offspring), while dead bacteria are discarded. At the end of one host generation (100 time steps), the reproduction of hosts is determined based on the survival probability in Eq. 1. Non-surviving hosts are discarded and each of the surviving hosts gives rise to a parent and offspring host as follows:

- The parent retains its current state (***x***, ***y***, **δ**) and the state of its bacterial population.
- The offspring host is created with properties defined by Eq. 5. Negative values of the sensitivity and detox are prevented by taking the absolute value of the outcome in Eq. 1. Each offspring receives a copy of the bacterial population of its parent (reflecting the state of the parent microbiome following 99 bacterial generations from the previous host replication). These populations are then iterated forward one bacterial generation and the surviving bacteria reproduce so as to define the initial state of the bacterial populations in the next host generation of the parent and its offspring.

## Additional file

Additional file 1: **Figure S1: (A)** Rapid selection of hosts with large **δ** under exposure to a pulse of toxin. **(B)** Host physiological stress over a host generation, versus time average of the bacterial carrying capacity. **(C)** Same as (B) for the time average of total bacterial detox versus bacterial carrying capacity. **Figure S2:** Distributions of physiological (***Ŝ***_***Ph***_) and toxic stress (***Ŝ***_***H***_) experienced by cloned parents and their offspring, following exposure to a toxin pulse. **Figure S3:** Average level of active toxin at the end of one host generation as a function of bacterial detox coefficient. **Figure S4**: Detox cost weakens the selection of hosts with more detox per bacterium while increasing the selection of hosts that accommodate more bacteria. **Figure S5**: The Lamarckian is not compromised by cost on bacterial detox. **Figure S6**: Temporal kinetics of phenotypic variability in response to toxic exposure. (DOCX 665 kb)
