# Supplementary material for: Darwinian selection of host and bacteria supports emergence of Lamarckian-like adaptation of the system as a whole
Source: Biol Direct. 2018 Oct 26;13:24. doi: 10.1186/s13062-018-0224-7 (PMC6889200; doi:10.1186/s13062-018-0224-7)
Supplement: Supplementary file 1 — Figure S1: (A) Rapid selection of hosts with large δ under exposure to a pulse of toxin. (B) Host physiological stress over a host generation, versus time average of the bacterial carrying capacity. (C) Same as (B) for the time average of total bacterial detox versus bacterial carrying capacity. Figure S2: Distributions of physiological (Ŝ Ph) and toxic stress (Ŝ H) experienced by cloned parents and their offspring, following exposure to a toxin pulse. Figure S3: Average level of active toxin at the end of one host generation as a function of bacterial detox coefficient. Figure S4: Detox cost weakens the selection of hosts with more detox per bacterium while increasing the selection of hosts that accommodate more bacteria. Figure S5: The Lamarckian is not compromised by cost on bacterial detox. Figure S6: Temporal kinetics of phenotypic variability in response to toxic exposure. (DOCX 665 kb) [file 13062_2018_224_MOESM1_ESM.docx]

**Supplementary Methods**:

***Cost on bacterial detox:*** To investigate the effects of detox cost to individual bacterium, we redefined the survival probability of bacteria as follows:

***(2’) P_B_ =* (*1 - N_B_ /2K_B_*) exp(-*S_B_*) exp(- *C_B_ y_B_*)**

where ***C_B_*** is a fixed coefficient providing a scale for the cost of detox secretion. The remaining components of the model were unchanged.

**Supplementary Figures:**


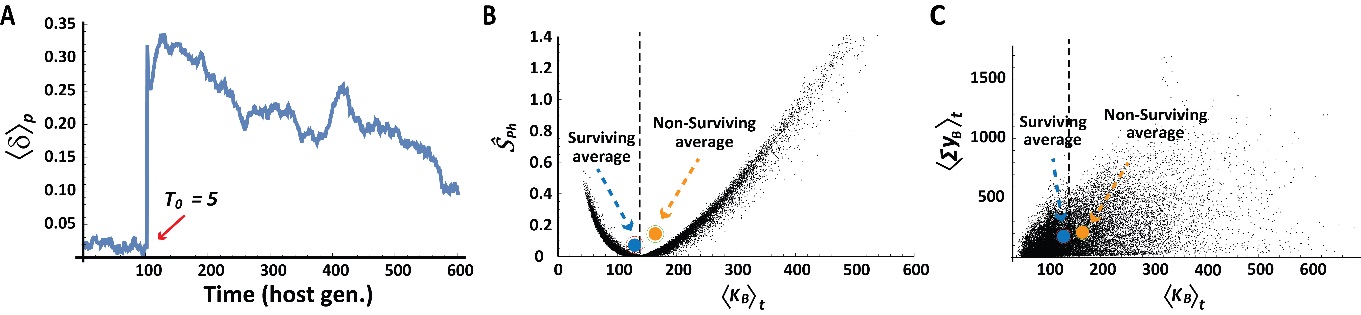


**Figure S1: (A)** Rapid selection of hosts with large **δ** under exposure to a pulse of toxin (***T_0_*** =5), applied at the initial time step, ***t*** ∈ [0, *Δ****t***] (red arrow). **(B)** Host physiological stress over a host generation, ***Ŝ _ph_* = *ln* (<*N_B_ > _t_ /K_B_^0^*) + (1 - <*N_B_ > _t_ /K_B_^0^*)**, versus time average of ***K_B_*** (Same as Fig. 1E, except for the exclusion of hosts which lost their bacteria). Blue and orange circles mark population averages for surviving and non-surviving hosts, respectively. Dotted line marks the ***K_B_*** value which minimizes the physiological stress. **(C)** Same as (B) for the time average of total bacterial detox versus bacterial carrying capacity.


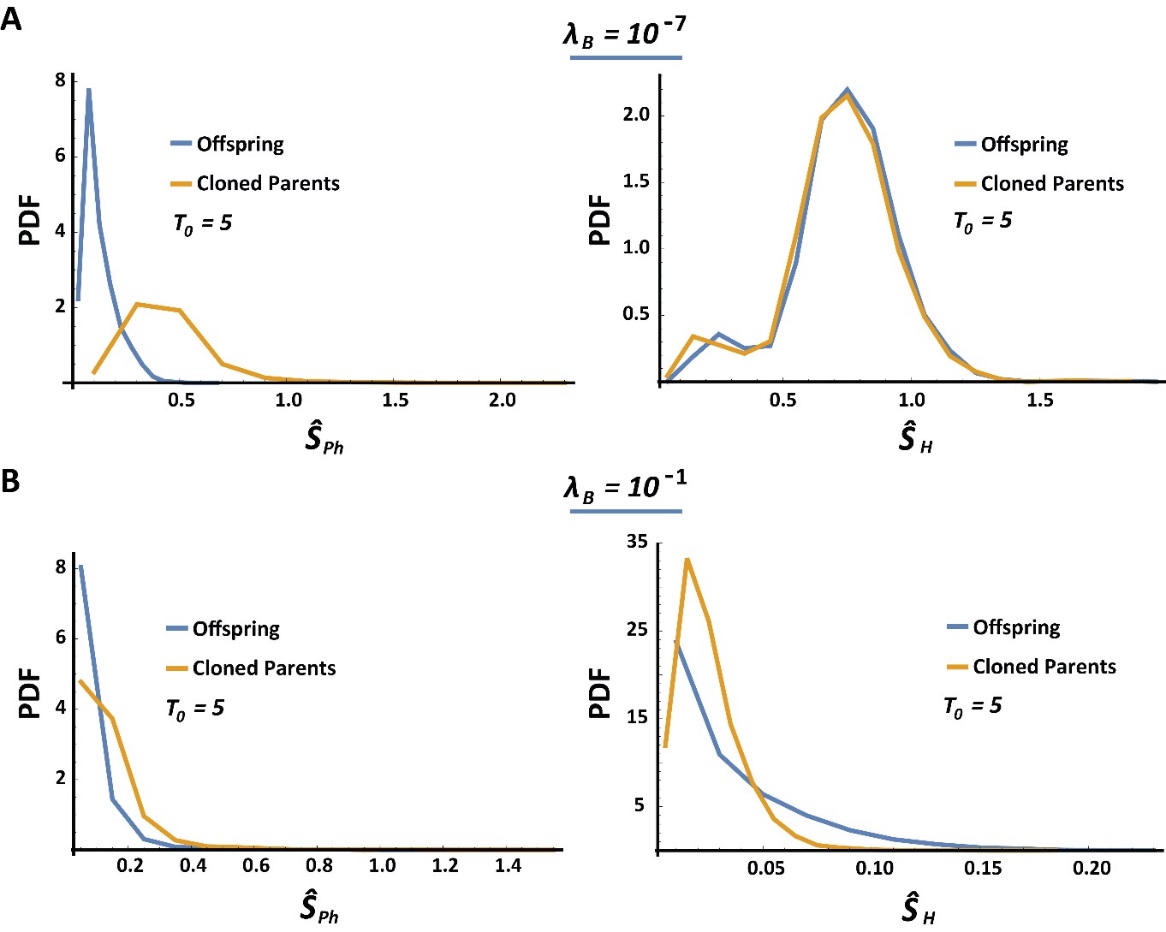


**Figure S2:** Distributions of physiological (***Ŝ _Ph_***) and toxic stress (***Ŝ _H_***) experienced by cloned parents and their offspring, following exposure to a toxin pulse (***T_0_*** =5) applied at the initial time step. **(A)** Case of low detox coefficient, **λ*_B_*** =10^-7^. **(B)** Case of high detox coefficient, **λ*_B_*** =10^-1^.


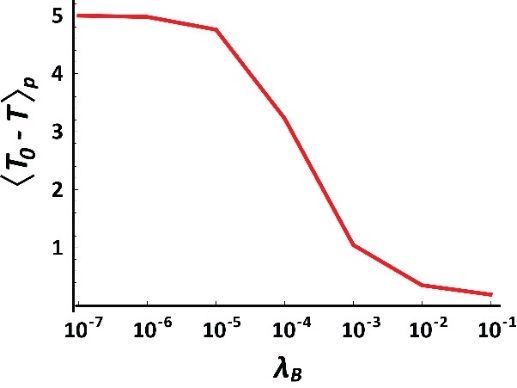


**Figure S3:** Average level of active toxin at the end of one host generation as a function of bacterial detox coefficient.


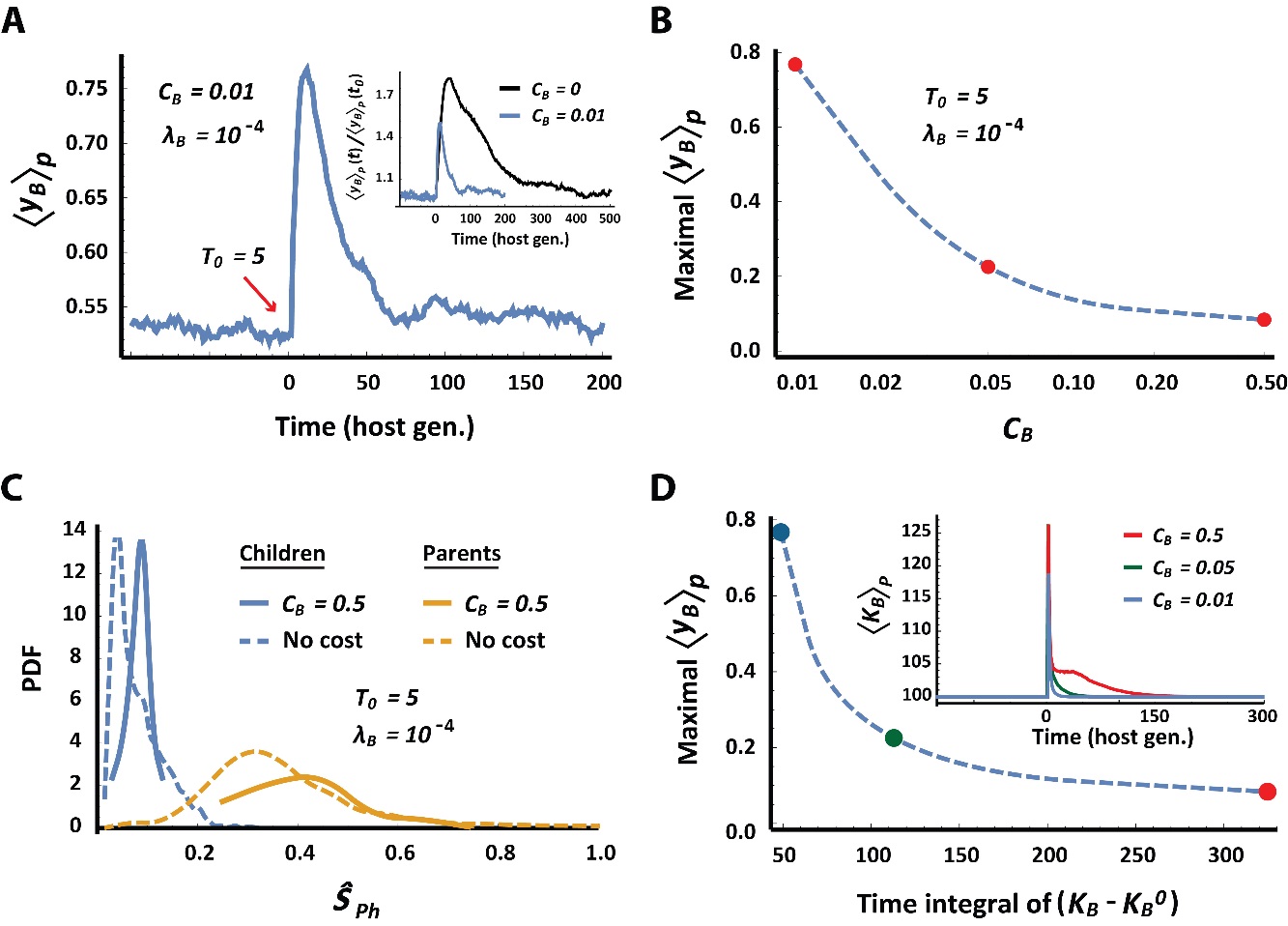


**Figure S4: Detox cost weakens the selection of hosts with more detox per bacterium while increasing the selection of hosts that accommodate more bacteria.** A population of hosts with vertically-transmitted bacteria was exposed to an influx of toxin, ***T_0_*** = 5, at *t_0_* = 0. **(A)** Temporal kinetics of the population average of detox secretion per bacterium, with a cost coefficient, **C*_B_*** = 0.01 and detox capacity of **λ*_B_*** =10^-4^. Inset displays the effect of detox cost (**C*_B_*** = 0.01 vs. **C*_B_*** = 0) on the change in the average detox relative to the respective levels at *t_0_* = 0. **(B)** Maximal level of detox secretion per bacteria that is achieved following selection with different choices of cost coefficients. **(C)** Distributions of the physiological stress (***Ŝ _Ph_***) to host offspring and their cloned parents (blue and yellow, respectively), with and without cost on detox secretion (solid vs. dotted lines). **(D)** Trade-off between selection of hosts with higher detox per bacterium and selection of hosts which accommodate more bacteria under exposure to toxin. The latter is indicated by integrated increase in ***K_B_*** over time and by monotonic, cost-dependent extension in the duration of selection for higher ***K_B_*** (inset).


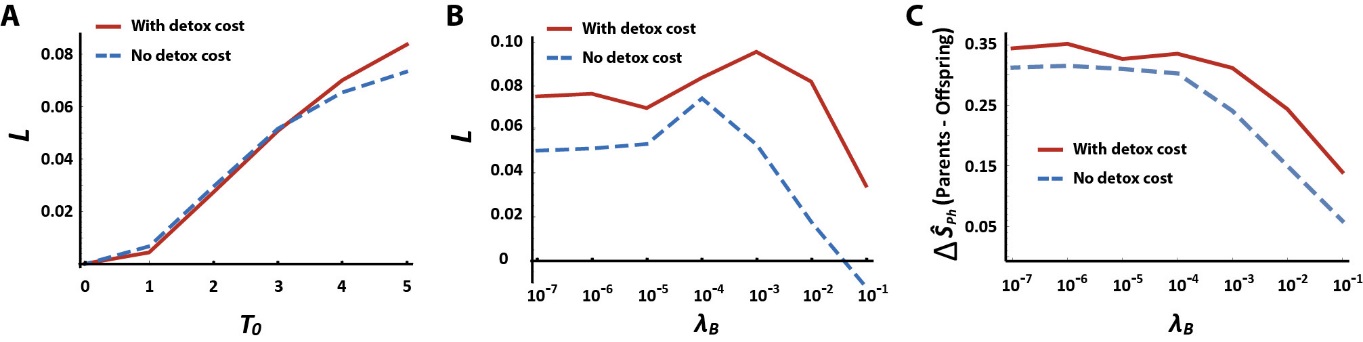


**Figure S5: The Lamarckian is not compromised by cost on bacterial detox. (A, B)** The Lamarckian as a function of toxin influx, ***T_0_*** (A) and detox capacity, **λ*_B_*** (B), with and without detox cost. **(C)** Effect of detox cost on the difference between cloned parents and their offspring with respect to the physiological stress, ***Ŝ _ph_***. Shown for different choice of **λ*_B_***.


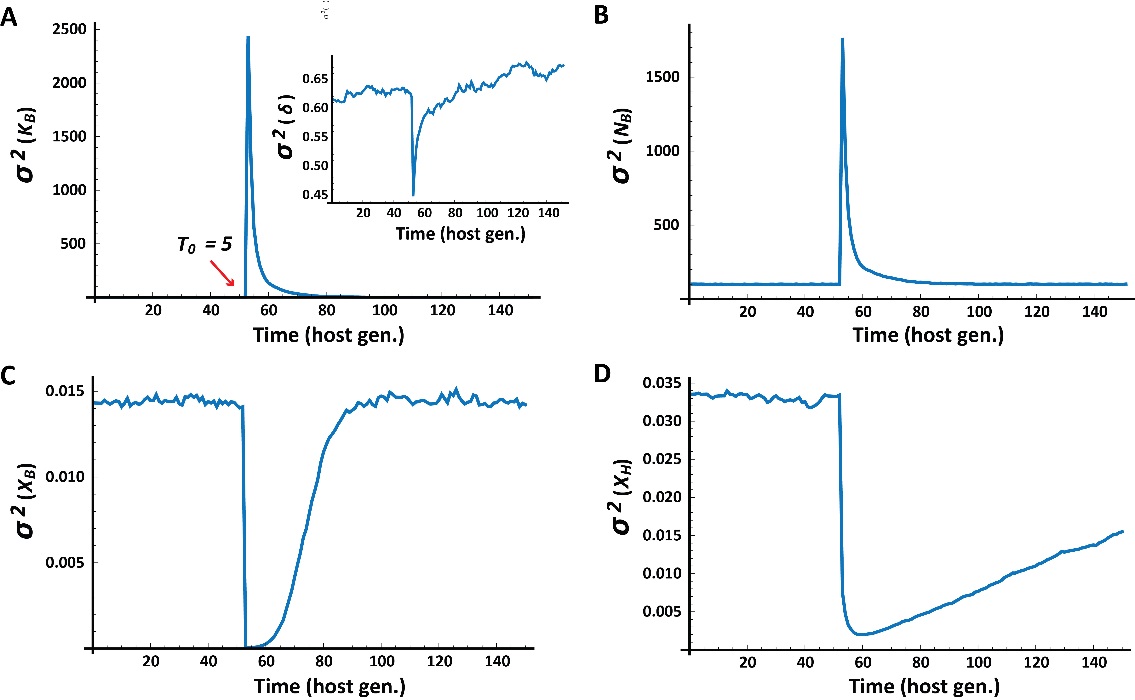


**Figure S6: Temporal kinetics of phenotypic variability in response to toxic exposure.** The population of host-microbiome systems is exposed to a toxin pulse (***T_0_*** =5) at generation 50 (red arrow in A). Shown are instantaneous variability between hosts with respect to: the bacterial carrying capacity (A), host **δ** (A, inset), size of the bacterial population (B), average sensitivity of resident bacteria (C) and average host sensitivity (D).
